# High Serum Level of IL-17 in Patients with Chronic Obstructive Pulmonary Disease and the Alpha-1 Antitrypsin PiZ Allele

**DOI:** 10.1155/2020/9738032

**Published:** 2020-01-30

**Authors:** Margarita Y. Pervakova, Alexandra V. Mazing, Sergey V. Lapin, Olga Y. Tkachenko, Anna I. Budkova, Elena A. Surkova, Vladimir L. Emanuel, Olga N. Titova

**Affiliations:** Pavlov First State Medical University of St. Petersburg, 197022 Saint Petersburg, Russia

## Abstract

Chronic obstructive pulmonary disease (COPD) is multifactorial disease, which is characterized by airflow limitation and can be provoked by genetic factors, including carriage of the PiZ allele of the protease inhibitor (Pi) gene, encoding alpha-1 antitrypsin (A1AT). Both homozygous and heterozygous PiZ allele carriers can develop COPD. It was found recently that normal A1AT regulates cytokine levels, including IL-17, which is involved in COPD progression. The aim of this study was to determine whether homozygous or heterozygous PiZ allele carriage leads to elevated level of IL-17 and other proinflammatory cytokines in COPD patients. *Materials and Methods*. Serum samples and clinical data were obtained from 44 COPD patients, who included 6 PiZZ, 8 PiMZ, and 30 PiMM A1AT phenotype carriers. Serum concentrations of IL-17, IL-6, IL-8, IFN-*γ*, and TNF-*α* were measured by the enzyme-linked immunosorbent assay (ELISA). All A1AT phenotypes were verified by narrow pH range isoelectrofocusing with selective A1AT staining. A turbidimetric method was used for quantitative A1AT measurements. *Results*. COPD patients with both PiZZ and PiMZ phenotypes demonstrated elevated IL-17 and decreased IFN-*γ* levels in comparison to patients with the PiMM phenotype of A1AT. Thereafter, the ratio IL-17/IFN-*γ* in PiZZ and PiMZ groups greatly exceeded the values of the PiMM group. Homozygous PiZ allele carriers also had significantly higher levels of IL-6 and lower levels of IL-8, and IL-6 values correlated negatively with A1AT concentrations. *Conclusions*. The presence of the PiZ allele in both homozygous and heterozygous states is associated with altered serum cytokine levels, including elevated IL-17, IL-17/IFN-*γ* ratio, and IL-6 (only PiZZ), but lower IFN-*γ* and IL-8.

## 1. Introduction

Chronic obstructive pulmonary disease (COPD) is currently the fourth leading cause of death in the world, a major cause of chronic morbidity and mortality [1]. The current pathophysiologic concept assumes COPD as complex disease with multifactorial background, based on the interaction of environmental and genetic factors [2]. The most well-studied predisposition factor for COPD is alpha-1 antitrypsin deficiency (A1ATD), which occurs as a result of carriage of pathogenic alleles of the Pi gene (SERPINA1, protease inhibitor) [3]. The most common and normally functioning A1AT allelic form is PiM, whereas the most abundant and clinically significant pathological allele is PiZ. About 95% cases of clinically manifested A1ATD occur as a result of the PiZZ phenotype [4]. Meanwhile, the heterozygous PiMZ phenotype leads to the so-called intermediate A1ATD [5] and is associated with increased risk of COPD, but mostly in ever-smokers [6]. The protease/antiprotease hypothesis explains the development of emphysema by the loss of A1AT ability to inhibit neutrophil proteases, mainly neutrophil elastase [7]. Recently, multiple immunomodulatory and anti-inflammatory A1AT functions were described, and several pulmonary and extrapulmonary pathologies, besides COPD, were found to be associated with A1ATD. In particular, A1AT suppresses NF-k*β* activation [8], reduces TNF-*α* expression [9, 10], and regulates TNF-*α* signaling [11]. Moreover, A1AT is capable of regulating the production of IL-1*β*, IL-6, and IL-8 [12] and lowering neutrophil chemotaxis by binding and inactivating IL-8 [13].

Neutrophilic inflammation in airways is considered central to the pathogenesis of COPD, regardless of the clinical phenotype, severity of disease, rapidity of lung function decline, and age of onset [14]. It was found that the proinflammatory cytokine IL-17 played a crucial role in COPD progression [15, 16]. This proinflammatory cytokine is essential in the acute phase of COPD, and it mediates COPD exacerbations and provokes bronchial hyperreactivity [17] and corticosteroid resistance [18]. Smoking patients demonstrated higher levels of IL-17 [15].

IL-17 is a key cytokine that is produced by type 17 T helper (Th17) cells and links T cell activation to neutrophil mobilization and activation [19]. IL-17 induces tissue inflammation mainly by stimulating the expression of several proinflammatory cytokines including IL-6, TNF-*α*, IL-1*β*, and IL-8 [20]. This leads to the expansion and accumulation of neutrophils in the focus of inflammation, as occurs, for example, in smoke-induced injury [21–23]. The formation of a Th17 cell subset is initiated by transforming growth factor- (TGF-) *β* together with the obligatory presence of IL-6 [24]. The production of Th17 is suppressed by IFN-*γ*, which is a proinflammatory cytokine with anti-inflammatory functions, and it possibly participates in resolution of inflammation [24–26]. The IL-17/IFN-*γ* ratio was suggested as a marker for prognosis and severity of inflammatory diseases [25, 27]. It was confirmed that A1AT also reduces Th17 cell formation, increasing the CD4+FoxP3+ Treg cell population, in contrast to IFN-*γ*, which stimulates Th1 formation [28].

Thus, both IL-17 and A1AT are very important molecules involved in COPD pathogenesis, and the modulatory A1AT effect on IL-17 secretion was confirmed. But there is still no evidence whether homozygous or heterozygous PiZ allele carriage modifies A1AT immunomodulatory properties and influence on IL-17 level in COPD.

The aim of this study was to evaluate whether homozygous or heterozygous PiZ allele carriage modifies the levels of IL-17 and other proinflammatory cytokines in COPD patients.

## 2. Materials and Methods

Serum samples and clinical data were collected from 44 male patients, who have suffered COPD exacerbations. The study design was approved by the local university ethics committee. Written informed consent was obtained from all study patients. Those patients included 14 PiZ allele carriers: 6 PiZZ and 8 PiMZ A1AT phenotypes. As far as normal PiMM phenotypes were identified in other 30 patients, their samples were used as a control group. All patients had mean age of 59.42 ± 11.32 years, and most of them were ex-smokers. They all demonstrated an FEV_1_ decline lower than 50% of the predicted value.

All A1AT phenotypes were identified by isoelectrofocusing, conducted in ultrathin agarose gels, containing 0.9% agarose IEF (GE Healthcare, USA), 8.7% glycerol (MP Biomedicals, USA), and 7.8% D-sorbitol (Sigma-Aldrich, USA). Narrow pH gradient was generated by adding ampholytes (pH 4.2-4.9) (GE Healthcare, USA) to the gel solution, cooled down to 60°C. Electrophoretic separation was performed with the Multiphor II Electrophoresis System (Pharmacia Biotech, Sweden). Serum samples were applied on gels with 3 *μ*l applicators. The prefocusing step was set at the following parameters: 450 V, 30 mA, 15 W, and 380 V/h. The focusing of A1AT molecules was performed at 1050 V, 28 mA, 21 W, and 1700 V/h. Since the separation was completed, the proteins were transferred to the Hybond-C extra membrane (GE Healthcare, USA) by western blotting under pressure of 2 kg for 10 minutes. The further steps included blocking by membrane incubation in 2.0% BCA solution, washing in saline buffer, and selective staining with horseradish peroxidase-conjugated goat anti-human A1AT antibodies (Bethyl Laboratories, Sweden). The resulting immune complexes were visualized by staining reaction with 3-amino-9-ethylcarbazole as a chromogenic substrate in the presence of hydrogen peroxide.

Quantitative A1AT measurements were performed in the samples with a turbidimetric commercial kit (Sentinel Diagnostics, Italy). Serum concentrations of IL-17, IL-6, IL-8, IFN-*γ*, and TNF-*α* were measured by the enzyme-linked immunosorbent assay (ELISA) with commercial kits (OOO «Cytokin», Russia).

The results are presented as median ± 75% interquartile range (IQR). Nonparametric data were compared by Kruskal-Wallis one-way analysis of variance. Dunn's pairwise multiple comparison posttest was used to compare each patient group. Correlations between the parameters were evaluated using Spearman's rank correlation test. Differences between the groups were considered significant at a *P* value of <0.05. Statistical analyses were performed with GraphPad Prism 6.0 (GraphPad Software, Inc., version for Windows 6.01).

## 3. Results

The following subgroups of COPD patients were analysed: 6 PiZZ, 8 PiMZ, and 30 PiMM phenotype carriers. Clinical and laboratory parameters of COPD patients with PiZZ, PiMZ, and PiMM phenotypes are presented in Table 1.

**Figure 1:**
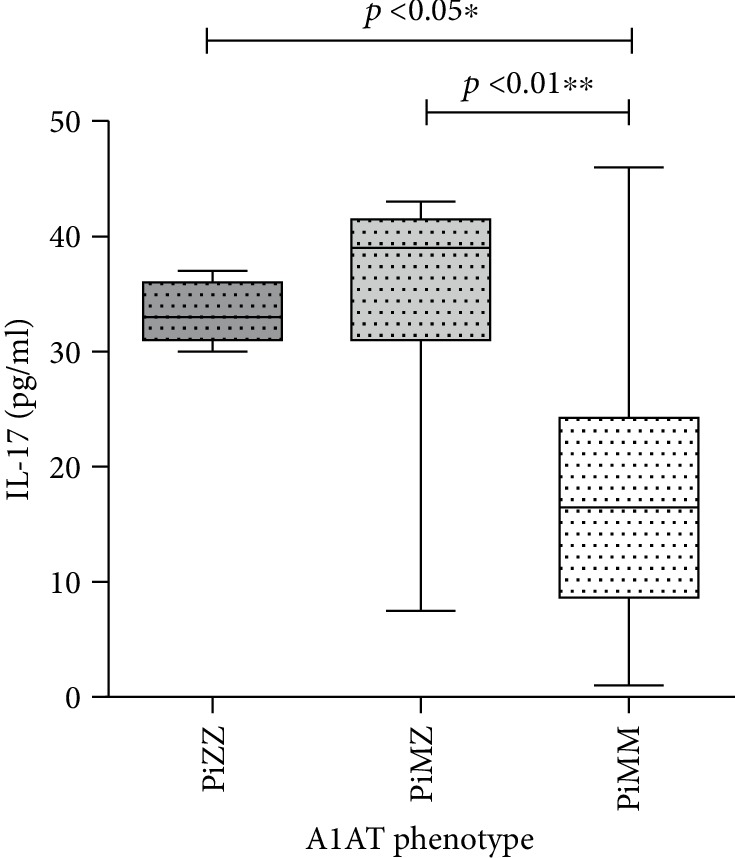
Comparison of IL-17 levels in COPD patients with PiZZ, PiMZ, and PiMM phenotypes of A1AT.

The median IL-17 level in patients with the PiZZ phenotype was 57.86 pg/ml (44.76-71.01 pg/ml), which was significantly higher than that in the normal PiMM phenotype: 1.44 pg/ml (1.24-1.81 pg/ml). The IL-17 level in PiMZ individuals was also elevated up to 82.39 pg/ml (37.87-121.8 pg/ml) (*P* < 0.01). The comparison of IL-17 levels in COPD patients with different A1AT phenotypes is presented in Figure 1.

On the contrary, the median level of IFN-*γ* was lower both in PiZZ (15.52 pg/ml (1.57-62.34 pg/ml)) and in PiMZ (15.98 pg/ml (5.67-22.0 pg/ml)) than in PiMM individuals (62.29 pg/ml (36.29-199.5 pg/ml)).

**Figure 2:**
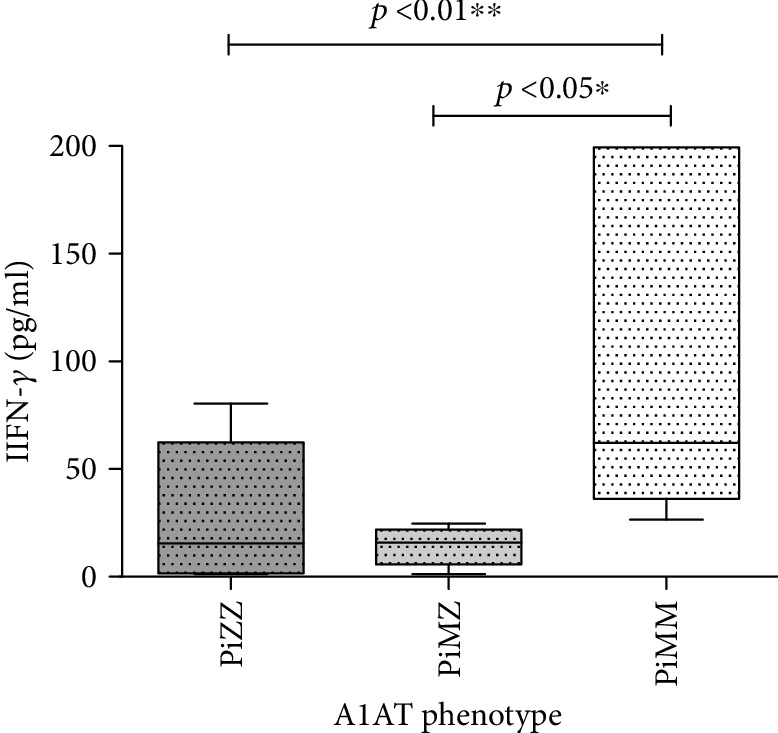
Comparison of IFN-*γ* levels in COPD patients with PiZZ, PiMZ, and PiMM phenotypes of A1AT.

The comparison of IFN-*γ* levels in COPD patients with different A1AT phenotypes is presented in Figure 2.

Consequently, the ratio of IL-17/IFN-*γ* was calculated as an integrative biomarker of disease severity. In both PiZZ and PiMZ groups, the IL-17/IFN-*γ* ratio exceeded the values of the PiMM group. The median IL-17/IFN-*γ* ratio value in patients with the PiZZ phenotype was 3.65 (2.38-36.6) and was significantly higher than that in the normal PiMM phenotype (0.026 (0.004-0.53)). The IL-17/IFN-*γ* ratio in PiMZ individuals was also elevated up to 6.81 (3.68-26.9).

**Figure 3:**
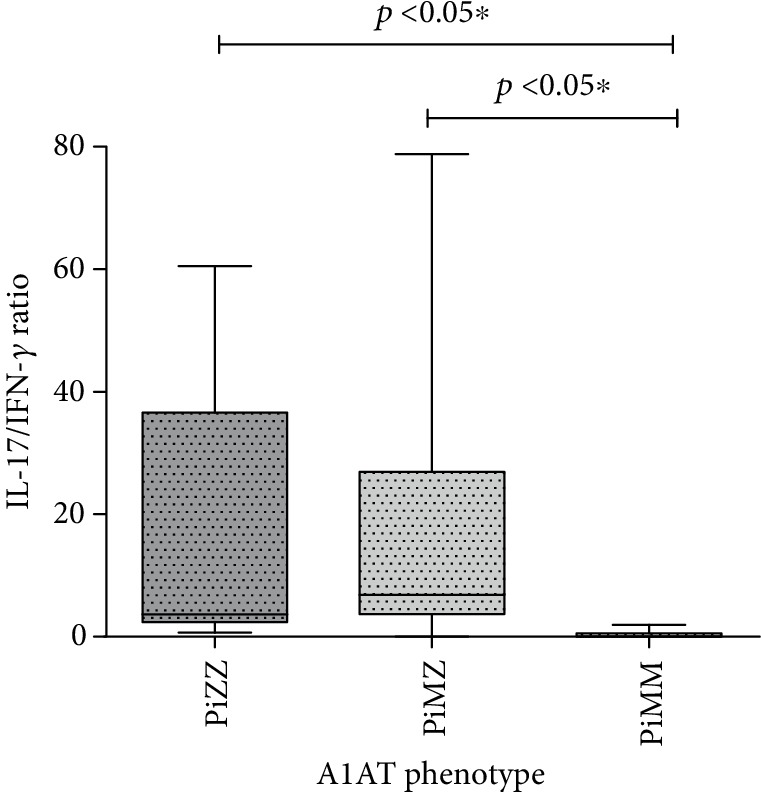
Comparison of IL-17/IFN-*γ* ratios in COPD patients with PiZZ, PiMZ, and PiMM phenotypes of A1AT.

The comparison of IL-17/IFN-*γ* ratios in COPD patients with different A1AT phenotypes is presented in Figure 3.

**Figure 4:**
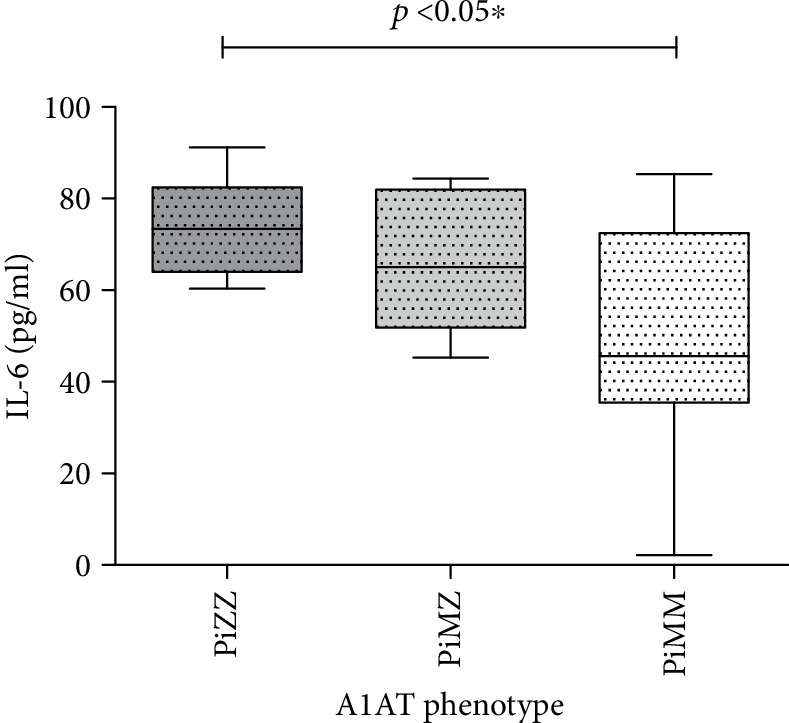
Comparison of IL-6 levels in COPD patients with PiZZ, PiMZ, and PiMM phenotypes of A1AT.

The median IL-6 level in PiZZ patients was also elevated up to 74.58 pg/ml (63.15-84.66 pg/ml), in comparison to PiMM (47.0 pg/ml (27.42-73.28 pg/ml)). It was not significantly increased in PiMZ patients (63.38 pg/ml (50.20-82.59 pg/ml)). The comparison of IL-6 levels in COPD patients with different A1AT phenotypes is presented in Figure 4.

**Figure 5:**
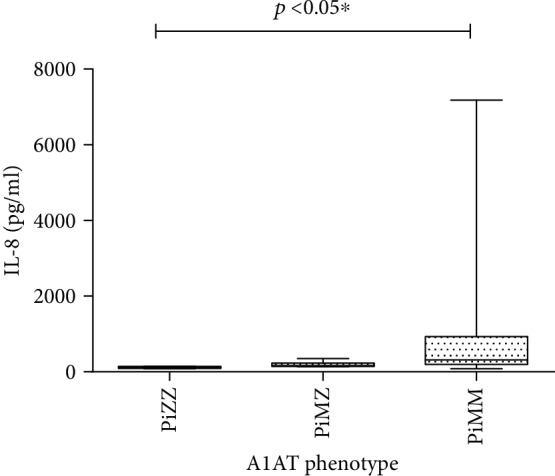
Comparison of IL-8 levels in COPD patients with PiZZ, PiMZ, and PiMM phenotypes of A1AT.

On the contrary, the median level of IL-8 was significantly lower in PiZZ (117.5 pg/ml (87.38-143.2 pg/ml)) versus PiMM (319.0 pg/ml (196.9-927.2 pg/ml)). The median level of IL-8 in PiMZ patients was 162.8 pg/ml (142.8-231.7 pg/ml), which was not significantly different. The comparison of IL-8 levels in COPD patients with different A1AT phenotypes is presented in Figure 5.

Both IL-17 and IL-17/IFN-*γ* ratio did not correlate with A1AT concentrations.

However, IFN-*γ* level showed a direct correlation with A1AT level, whereas IL-6 level correlated negatively with A1AT concentration. IL-8 showed a weak positive correlation with A1AT. The results of correlation analysis between A1AT concentrations and proinflammatory cytokine levels are presented in Table 2.

Serum TNF-*α* levels of all individuals except for two PiZZ and PiMM phenotype carriers were below the lower detection limit of assay (1 pg/ml).

## 4. Discussion

Immunomodulatory and tissue-protective properties of A1AT were described by many researchers during the last decade. It was proven that PiZ allele carriage reduces A1AT adhesion to neutrophil elastase and other proteases, leading to alveolar surfactant, elastin, and extracellular matrix degradation [29]. Regarding immunomodulatory properties, it was also reported that COPD patients with A1ATD had higher TNF-*α*, IL-6, IL-1*β*, and IL-8 production in comparison to COPD patients without A1ATD [12, 13, 30]. Meanwhile, we did not find any data about IL-17 and IFN-*γ* levels in COPD patients with A1ATD in comparison to patients with the PiMM phenotype of A1AT.

This study was aimed at investigating whether PiZ allele carriage influences IL-17 and other proinflammatory cytokine serum levels in COPD. The following COPD patient groups were analysed: 6 PiZZ, 8 PiMZ, and 30 PiMM A1AT phenotype carriers. Patients with the PiZZ phenotype were younger and had a more intensive FEV_1_/FVC ratio decline, leading to faster obstruction development. It was also noted that PiZZ patients had higher hemoglobin and hematocrit values (*P* < 0.05), which indicate secondary erythrocytosis because of respiratory failure in COPD. Patients, who were PiMZ carriers, demonstrated slightly lower A1AT level and a lower FEV_1_/FVC ratio as compared to controls.

Patients with COPD and also with the PiZZ and PiMZ phenotype of A1AT demonstrated significantly increased IL-17 serum level and decreased IFN-*γ* level in comparison to PiMM patients. Both IL-17 and IFN-*γ* are proinflammatory cytokines, but only IFN-*γ* also exhibits some anti-inflammatory properties [31] and is possibly involved in a resolution of inflammation. This is confirmed by the observation that when inflammation subsides, the expression of IFN-*γ* remains at relatively high level despite the prominent decrease in the expression of other proinflammatory cytokines [26]. This may be related to Th17 ability to switch the production from IL-17 to IFN-*γ* [25]. Consequently, we calculated the IL-17/IFN-*γ* ratio, which was proposed by some researchers as an unfavorable marker of inflammatory disease progression [25, 27]. In this study, the IL-17/IFN-*γ* ratio was significantly elevated in both homozygous and heterozygous PiZ allele carriers.

In PiZZ patients, the IL-6 level was significantly higher than that in PiMM patients, though in PiMZ patients, it was elevated insignificantly. Moreover, IL-6 showed an invert correlation with A1AT concentrations. This finding confirms a hypothesis that in inflammation A1AT regulates IL-6 expression negatively and low A1AT level results in elevated IL-6 level. IL-6 is a proinflammatory cytokine and an acute phase mediator, which is essential for Th17 differentiation, and IL-17 expression increases further synthesis of IL-6 [24]. Moreover, IL-6 together with TNF-*α* is considered a mediator of systemic COPD effects (cachexia, skeletal muscle dysfunction, cardiovascular diseases, depression, hypodynamia, and osteoporosis), leading to an unfavorable outcome [32].

Serum IL-8 level in homozygous PiZ allele carriers was lower than that in PiMM phenotype carriers. However, many researchers report an increased IL-8 activity in sputum in both PiZZ and PiMZ phenotype carriers [13, 30]. Such elevation of local, but not systemic, IL-8 concentration can possibly be explained by higher consumption and local airway production of IL-8, stimulated by circulating IL-17 [33].

Undetectable TNF-*α* levels were found in most study patients. A similar result was reported by Vernooy et al. who have suggested the different regulations of inflammation in the pulmonary and systemic compartment [34].

## 5. Conclusions

The presence of the PiZ allele in both homozygous and heterozygous states in COPD patients presumably affects A1AT immunomodulatory properties, leading to higher IL-17 and IL-17/IFN-*γ* ratio and lower IFN-*γ*. Homozygous PiZ allele carriers also have higher IL-6 levels, but lower IL-8. These changes predict an unfavorable outcome and may identify new potential targets for therapy in COPD patients.

## Figures and Tables

**Table 1:** Clinical and laboratory parameters of groups of COPD patients, divided by the A1AT phenotype.

A1AT phenotype	PiZZ	PiMZ	PiMM	*P*
A1AT concentration (mg/l)	310.00 (191.50-463.30)	1089.00 (848.00-1464.00)	1209.00 (1084.00-685.00)	ZZ/MZ: *P* < 0.05 ZZ/MM: *P* < 0.001
Age (year)	47.50 (41.25-58.0)	64.50 (57.0-70.25)	64.50 (58.50-65.50)	ZZ/MZ: *P* < 0.05 ZZ/MM: *P* < 0.05
FEV_1_ (% predicted)	25.78 (20.35-7.76)	35.64 (24.19-41.62)	27.82 (25.35-38.91)	ns
VLC (% predicted)	61.36 (56.23-6.72)	58.17 (51.66-74.65)	50.12 (40.75-66.55)	ns
FEV_1_/FVC (% ratio)	30.20 (23.30-1.59)	42.72 (33.59-45.85)	48.25 (40.58-64.26)	ZZ/MZ: ns ZZ/MM: *P* < 0.05
RBC count (×10^9^/l)	5.36 (5.08-5.79)	4.88 (4.27-5.43)	4.63 (4.36-5.19)	ns
Hemoglobin (g/l)	160.50 (149.80-174.80)	137.50 (128.00-54.80)	151.00 (141.50-158.00)	ZZ/MZ: *P* < 0.05 ZZ/MM: *P* < 0.05
Hematocrit (%)	46.30 (44.0-56.55)	40.40 (38.08-42.55)	42.90 (40.60-45.75)	ZZ/MZ: *P* < 0.05 ZZ/MM: *P* < 0.05
WBC count (×10^9^/l)	7.95 (5.16-12.03)	10.38 (8.60-15.7)	12.97 (8.40-16.94)	ns
Ever-smokers/never-smokers	3/3	8/0	28/2	ns

All quantitative data are presented as median ± 75% interquartile range (IQR). COPD: chronic obstructive pulmonary disease; A1AT: alpha-1 antitrypsin; FEV_1_: forced expiratory volume in one second; FVC: forced vital capacity; RBC: red blood cells; WBC: white blood cells.

**Table 2:** Correlation analysis between A1AT concentrations and proinflammatory cytokine levels in patients with COPD.

	A1AT concentration (mg/l)	
	*r*	*P*
IL-17 (pg/ml)	-0.029	ns
IFN-*γ* (pg/ml)	0.5191	<0.05
IL-17/IFN-*γ*	-0.4262	ns
IL-6 (pg/ml)	-0.5678	<0.01
IL-8 (pg/ml)	0.4534	<0.05

COPD: chronic obstructive pulmonary disease; A1AT: alpha-1 antitrypsin.

## Data Availability

The data used to support the findings of this study are available from the corresponding author upon request.
